# The novel manualized RELIEVE-group treatment for burdened relatives of cancer patients: a feasibility study

**DOI:** 10.3389/fpsyg.2024.1492219

**Published:** 2025-01-03

**Authors:** Jessica Neumann, Jil Beckord, Helen Samira Hesse, Carl Martin, Carlotta Mons, Diana Chur, Jörg Hense, Mitra Tewes, Martin Teufel, Eva-Maria Skoda

**Affiliations:** ^1^Clinic for Psychosomatic Medicine and Psychotherapy, University of Duisburg-Essen, LVR-University Hospital Essen, Essen, Germany; ^2^Center for Translational Neuro- and Behavioral Sciences (C-TNBS), University of Duisburg-Essen, Essen, Germany; ^3^West German Cancer Center, Department of Medical Oncology, University Duisburg-Essen, Essen, Germany; ^4^German Cancer Consortium (DKTK), Partner Site University Hospital Essen, Essen, Germany; ^5^West German Cancer Center, Department of Palliative Medicine, University Hospital Essen, University of Duisburg-Essen, Essen, Germany

**Keywords:** relatives, cancer, distress, depression, anxiety, treatment, feasibility

## Abstract

**Introduction:**

The experience of cancer among relatives is characterized by an increase in anxiety and depression, stress, and a reduction in quality of life. However, there is a paucity of psychosocial support programmes for relatives and a dearth of evidence-based, manualized interventions. Accordingly, the present study aims to assess the acceptability, defined as participant drop-out and satisfaction, and feasibility, in terms of mental health improvement, of a novel manualized psycho-educational group intervention.

**Methods:**

The manual was developed on the basis of previous research into psychotherapy. A total of 33 relatives of cancer patients were recruited from the West German Tumor Center and participated in the five modules of the RELIEVE treatment which included an introduction, communication skills, stress and anxiety management and self-care. The primary outcome was assessed using a range of measures, including anxiety (GAD-7), depression (PHQ-8), stress (PSQ), self-efficacy (SES), need for support (SCNSP&C-G), and quality of life (WGOQOL-BREF) before and after the completion of the treatment programme. A paired samples T-test was employed to assess the feasibility of the treatment, with pre- and post-scores being compared. The secondary outcome of treatment acceptance was evaluated by calculating the drop-out rate and scoring a treatment satisfaction questionnaire.

**Results:**

A high level of satisfaction was reported by participants. The drop-out rate for the treatment was minimal, at only 2.86%. Following the completion of the treatment programme, there were significant improvements in anxiety, depression and stress scores, as well as an improvement in quality of life. No significant improvements were observed in self-efficacy, work and social security needs, and quality of life in the social relationships domain.

**Discussion:**

The RELIEVE intervention demonstrated high feasibility and acceptance among emotionally affected relatives of cancer patients, addressing a gap in previous interventions that were often limited in scope and lacked standardised manuals.

**Conclusion:**

This feasibility study on treatment acceptance underlines the importance of measures that are tailored to the specific needs of cancer patients’ relatives, and of integrating them into the general healthcare system.

## 1 Introduction

Approximately 500.000 new cases of cancer are registered each year in Germany (Katalinic et al., 2023). Cancer is a disease widely feared due to its high mortality rate and severe physical consequences, affecting not only patients but also posing intense psychosocial challenges for their closest social network of relatives, thereby rendering cancer a “we-disease” (Ünsar et al., 2021; Kleine et al., 2019). In Germany, the term “relative” is defined as family members (i.e., parents, spouses, children) or another individual with a strong social connection to the patient, such as a close friends or partner (Deutsche Krebsgesellschaft, Deutsche Krebshilfe, AWMF, 2020). For these relatives, the challenges include increased caregiving responsibilities, feelings of uncertainty and fear of loss, often compounded by a lack of adequate support, compensation, or respite (Gray et al., 2019). Many experience significant psychological distress—anxiety, depressive symptoms, and stress—that diminishes their quality of life (Mahendran et al., 2017). It is estimated that between 25 and 40% of relatives of cancer patients experience anxiety and depressive symptoms at some point throughout the course of the disease (Oechsle et al., 2019). These symptoms undergo changes throughout the progression of the disease, reaching their peak during the palliative stage (Götze et al., 2016). Furthermore, between 55 and 90% of relatives also experience clinically relevant psychological stress, which has a detrimental impact on their physical and mental well-being (Oechsle et al., 2019; Priya et al., 2021).

The most commonly reported stressors for relatives include uncertainty about disease progression, sleep deprivation, and financial insecurity (Ilic et al., 2023). The quality of life (QoL) of family members is known to be significantly affected by their role as carers (Cai et al., 2021). Relatives are inclined to prioritize the well-being of the patient over their own, and the demands of caregiving can result in interference with work or educational responsibilities (Turchi et al., 2022a). The extent of care work and emotional distress can lead to a loss of private social connections (Cai et al., 2021). Viewing health as a continuum underscores that these challenges extend beyond immediate reactions to a loved one’s illness, reflecting a broad spectrum of stressors affecting the overall well-being of relatives (Turchi et al., 2022b). The burden of caregiving is frequently overlooked, and the healthcare system often fails to meet the demand for support from relatives of cancer patients, as most interventions up to date are not specifically tailored to their emotional and psychosocial needs. Therefore, in order for the healthcare system to meet the demands, the development of new targeted interventions is required to directly address the specific emotional needs of patient relatives.

The lack of control that relatives experience when facing the progression of an illness can result in feelings of helplessness and disempowerment, which are associated with a low sense of self-efficacy (Badger et al., 2010). In this context, self-efficacy refers to the relative’s perceived ability to cope with the demands of the illness and other challenges such as fulfilling caregiving responsibilities and coping with the emotional impact of a loved one’s illness (Hebdon et al., 2021; Kershaw et al., 2015). The concept of self-efficacy has been demonstrated to predict patient health outcomes by improving the quality of caregiving activities as well as the health outcomes of caregivers themselves (Kershaw et al., 2015). For relatives of cancer patients, self-efficacy—the belief in one’s ability to handle challenging situations—is essential as it boosts, resilience, motivation, and coping abilities in caregiving, benefiting both their own and the patient’s quality of life (Hendrix et al., 2016). Relatives have been found to report a need for professional support in order to cope with uncertainty, fear of recurrence, sadness and maintaining an optimistic outlook (Rosenberger et al., 2012). The provision of social support and additional forms of assistance, such as counselling, have been demonstrated to enhance personal resources, including self-efficacy (Astrup et al., 2020). However, it is notable that the majority of research exploring the mental wellbeing of relatives has centred on informal caregivers of cancer patients, with less focus on relatives who may engage in fewer caregiving activities but are more profoundly emotionally affected (Lee et al., 2015).

Psychoeducation, providing caregivers with information on for example caregiving, self-care, and coping strategies, and group therapy have been identified as promising interventions for relatives of cancer patients. A meta-analysis of the literature revealed that psychoeducation, aimed at increasing the preparedness of caregivers and meeting their emotional and psychosocial needs, was associated with improvements in caregiver burden, anxiety, depression, self-efficacy, physical health, and QoL among caregiving relatives (Bilgin and Özdemir, 2022; Cheng et al., 2022; Kusi et al., 2022). However, most existing studies focus on the short-term effects of psychosocial interventions for relatives of cancer patients, while the long-term effectiveness of these interventions has only been studied to a limited extent. This lack of data leaves unanswered the question of whether improvements in well-being, anxiety management or quality of life persist over time (Cheng et al., 2022). Furthermore, meta-analyses indicate that the rate of withdrawal from treatment varies greatly between studies (0–71.4%). The most common reason for dropouts was death of cancer patients (Bilgin and Özdemir, 2022; Cheng et al., 2022). A comparison of intervention types revealed that psychoeducation was more effective than anticipatory guidance interventions (Thompson and Young-Saleme, 2015). Furthermore, the efficacy of psychoeducation was enhanced when combined with cognitive-behavioral and mindfulness-based approaches (Lei et al., 2023). Many existing programmes concentrate on supporting the practical caregiving role without specifically aiming to foster self-efficacy and resilience among relatives—key factors for coping with long-term emotional challenges (Hendrix et al., 2016). Given that relatives often perceive a sense of isolation in navigating their struggles, group-interventions can serve as a space where for relatives to interact with others facing similar experiences. This effect is most pronounced when group-interventions incorporate group activities (Gray et al., 2019). Nevertheless, in comparison to individual interventions, group formats demonstrated comparatively limited efficacy in enhancing marital functioning (Jones et al., 2013), as well as in alleviating depressive and anxiety symptoms (Lei et al., 2023). It is noteworthy that a considerable proportion of previously investigated intervention strategies have not been tailored to the specific needs of relatives, and there is a scarcity of standardized manuals (Kleine et al., 2019). In addition, most psychological support services for relatives of cancer patients are not yet systematically integrated into the general healthcare system and often remain limited to clinical facilities or specialized centres (Gray et al., 2019).

To reduce the psychological distress of cancer patient relatives, we have developed an innovative manualized psychoeducational group intervention called the RELIEVE intervention. RELIEVE consists of widely used, evidence-based treatment principles tailored to the specific needs of patient relatives and fills the aforementioned gap in the healthcare system. The programme is structured and outlined in a detailed, user-friendly manual, which includes a brief introduction for the user, explaining the purpose and scope of the programme and its’ sessions. Additionally, predefined materials in form of worksheets for the participants are available for each session. RELIEVE includes content and coping strategies relevant to various cancer stages—such as curative, chronic, and palliative phases – and types. The modules cover stress management, resilience-building, and communication skills that can be adapted to the emotional demands encountered both in early disease management and in end-of-life care. RELIEVE explicitly considers the varied relationships and responsibilities among cancer patient relatives. Existing interventions often focus on primary caregivers, neglecting the unique burdens experienced by siblings, children, friends, and distant relatives. RELIEVE’s structure incorporates tailored strategies that address the specific needs of different family roles and relational contexts, fostering a support network that reflects the diverse experiences of relatives. The distinct advantage of our manual and associated intervention is its universal applicability. Unlike approaches tailored to specific patient populations, our resources are designed to support patient relatives of patients across all types and stages of cancer. Additionally, the intervention is targeted at not only close family members but also close friends or caregivers who are involved in the care of the cancer patient and/or have a close emotional connection to the cancer patient, and who feel emotionally affected by their caregiving role and the illness of the patient. This broad applicability ensures that caregivers can benefit from our intervention regardless of the specific cancer diagnosis or progression stage, making it a versatile tool in the realm of oncology support. The present study aims to test the acceptability and feasibility of the RELIEVE intervention. Regarding acceptability, the study poses the following first research question (RQ1): Do relatives adhere to the RELIEVE intervention and report satisfaction with the treatment upon completion? It is hypothesized (H1) that relatives report high levels of treatment satisfaction and that there will be a low dropout rate. To test the feasibility of the intervention, the following second research question (RQ2) is as follows: Is the RELIEVE intervention effective in improving anxiety, depression, stress, QoL, self-efficacy, and need for support among cancer patients relatives? It is hypothesised (H2) that the intervention reduces anxiety, depression, stress, and need for support and increases QoL and self-efficacy when comparing pre- and post-treatment scores.

## 2 Methods

### 2.1 Participants

Relatives (family members, partners, and close friends) of cancer patients were recruited at the West German Tumor Centre through screening tools such as the electronic Psycho-Oncological Screening (ePOS), patient newsletters, other digital channels (websites, social networks), and via direct contact by personal staff. As part of the patient survey in ePOS, one specific question addresses whether the patient’s relatives feel burdened and need support. If this is affirmed, caregivers receive information on available services and studies. In order to be included in the study, participants had to be between 18 and 80 years old and have completed the informed consent form. Exclusion criteria were: Unstable psychopathological states (e.g., suicidality, psychotic symptoms), severe cognitive or physical impairments, an age of >80 years to minimise comorbidity with mobility impairments, and insufficient level of the German language. Initially, 35 participants started the intervention programme, with one participant dropping out during the intervention (2.86%) and one dropping out at post-treatment assessment, resulting in an overall drop-out rate of 5.71%. The final sample consisted of 33 participants (81.8% women, 18.2% men) with a mean age of 48.79 years (*SD* = 14.87).

### 2.2 Procedure

The study was approved by the Ethics Committee of the Medical Faculty of the University of Duisburg-Essen (22-10754-BO). Participants gave their written informed consent. They completed a series of questionnaires at baseline before the intervention was delivered. The intervention started two weeks after the baseline measurements and lasted five weeks, with one group therapy session per week. At the end of the intervention, participants responded to the same set of questionnaires as at baseline.

### 2.3 “RELIEVE” intervention

The intervention manual was developed based on established psychotherapy research in cognitive-behavioral therapy (CBT), acceptance and commitment therapy (ACT), and mindfulness-based approaches, forming the foundation for both the manual itself and the group implementation. The RELIEVE psychoeducational group intervention included five modules that utilized CBT techniques to address negative thought patterns and improve stress management. Mindfulness-based approaches were integrated to enhance emotional regulation and reduce burnout, while ACT strategies helped participants accept challenging emotions and pursue value-oriented living. Additionally, systemic therapy principles were applied to improve family communication and clarify roles within the family system (Faller et al., 2013; Jones et al., 2013; Kusi et al., 2022; Rush and Sharma, 2016). The group sessions, based on these therapeutic approaches, included case examples, sharing of personal experiences, exercises, and worksheets. A detailed description of the full programme is provided in Table 1.

**Table 1:** Overview of the topics, contents and exercises of RELIEVE.

	Goals	Intervention methods
Introduction (Module 1)	Introduction round Group rules Defining treatment goals Discussing wishes/fears	Information/Organisation: Worksheet: treatment goals, group rules Self-observation, wishes: Worksheet: Mindmap (wishes vs. fears) Mindfulness Imagination exercise: “baggage” Resource cards
Stress (Module 2)	Mastering stressful situations Identifying resources	Psychoeducation Learning about the meaning of thoughts for subjective experience and feelings of stress Worksheets: Lazarus Stress Model, Vulnerability-Stress Model Technique to cope with stress Mindfulness Body Scan Resource cards
Communication (Module 3)	Identifying possible problems within family, partnership, or treatment-specific communication. Learning skills for successful communication	Psychoeducation Worksheets: Four-Sides Model. Sender-Receiver-Model. Social Skills Training Exercise: Role play Mindfulness Imagination exercise: “Safe” Resource cards
Anxiety (Module 4)	Analyzing personal anxiety structures Learn how to deal with stressful feelings	Psychoeducation Worksheets: Vicious Cycle of Fear Situations that cause anxiety Functionality of the anxiety system Mindfulness Breathing exercise Resource cards
Self-care (Module 5)	Enhancing self-efficacy and self-compassion Reflecting on RELIEVE treatment	Psychoeducation Self-esteem and its importance in the context of cancer Worksheet: self-efficacy healthy vs. unhealthy Exercise: emergency kit with individual helpful skills Exercise: Strengthen my Self-Esteem House Worksheet: Resources to strengthen resilience Mindfulness Favorite imagination exercise Resource cards Evaluation of RELIEVE

### 2.4 Measures and psychometric instruments

Treatment satisfaction was assessed using the German version (Schmidt et al., 1989) of the Client Satisfaction Questionnaire (CSQ), originally developed by Attkisson and Zwick (1982). The scale consists of eight items, ranging from 1 = “poor” to 4 = “excellent.” The total score can range from 8 to 32, with ≥23 indicating good treatment satisfaction. The CSQ is highly reliable and has been widely used in recent research (Bodschwinna et al., 2022; Willems et al., 2019).

The German version (Löwe et al., 2008) of the Generalised Anxiety Disorder Scale-7 (GAD-7; Spitzer et al., 2006) was used to assess generalised anxiety symptoms in the past two weeks. The questionnaire consists of seven items that are rated on a scale from 0 = “not at all” to 3 = “nearly every day.” The ratings are calculated into one total score that can range from 0 to 21 with a total score of 0–4 indicating no generalised anxiety, 5–9 indicating mild generalised anxiety, 10–14 moderate generalised anxiety, and 15–21 severe generalised anxiety. The German version is validated and a well-known tool that is frequently used in research and clinical settings (Hinz et al., 2017).

The German version (Löwe et al., 2002) of the Patient Health Questionnaire Depression Scale-8 (PHQ-8; Kroenke et al., 2009) was used to measure depressive symptoms in the past two weeks. It consists of eight items that are rated on a 4-point scale ranging from 0 = “not at all” to 3 = “almost every day.” All items are combined into a total score that can range from 0 to 24. A score of ≥10 indicates major depression and a score of ≥20 indicates severe major depression. The German translation has been validated and is widely used.

Stress was assessed using the validated German translation (Fliege et al., 2001) of the Perceived Stress Questionnaire (PSQ; Levenstein et al., 1993). The PSQ aims to capture the subjective perception of stress as well as the subsequent processing of stressors. The 30 items are rated on a scale from 1 = “hardly ever” to 4 = “usually.” The questionnaire consists of seven scales, namely: overload, irritability, harassment, joy, fatigue, worry and tension. The PSQ index score ranges from 0 to 1, with a higher score indicating greater stress.

QoL was assessed using the German translation (Skevington et al., 2004) of the abbreviated Quality of Life questionnaire (WHOQOL-BREF; Whoqol Group, 1998). The instrument captures participant’s self-reported QoL over the past two weeks in four health domains (physical health, psychological health, social relationships, environment) with 24 items, ranging from 1 = “not at all” to 5 = “completely.” The QoL in each domain is assessed with domain-specific total scores that can range from 4–20.

The German version of the General Self-Efficacy Scale (SES; Schwarzer and Jerusalem, 1999) was used to measure participants’ general sense of self-efficacy. The scale consists of ten items, ranked on a 4-point Likert scale, and has been widely used for over 20 years. All items are summed to give a total score.

The German version (Sklenarova et al., 2015) of the Supportive Care Needs Survey for Partners and Caregivers (SCNSP&C-G; Girgis et al., 2011) was used to measure the need for support of relatives of cancer patients in the last month. The SCNSP&C-G consists of 45 items that are rated on a 5-point scale. The questionnaire covers four subscales: healthcare-service needs, psychological and emotional needs, work and social security needs, and communication and family needs. A higher score indicates a greater need for support. The German translation has been validated and is a common screening tool in clinical and research settings.

### 2.5 Data analysis

To test H1, the dropout rate and the mean CSQ scores were calculated. To test H2, 12 paired samples t-tests were calculated to compare pre- and post-treatment scores. The GAD-7, PHQ-8, PSQ, the four WHOQOL-BREF domains (Physical Health, Psychological Health, Social Relationships, Environment), SES, and the four SCNSP&C-G domains (Healthcare-Service Needs, Psychological and Emotional Needs, Work and Social Security Needs, and Communication and Family needs) were used as outcome variables. To avoid Type 1 Error due to multiple testing, the alpha level of *α = 0*.05 was adjusted using the Bonferroni method (adjusted *α* = 0.05/12 = 0.004). Cohen’s *d* values were interpreted using the thresholds suggested by Sullivan and Feinn (2012). Variables representing the difference between time points were generated to test assumptions for the paired samples t-test. There were no missing values in any of the variables. Outliers were examined using boxplots. Outliers were found in the QoL difference scores in the domains of Psychological Health and Environment, in the SES difference score, and in the SCNS difference scores in the domains of Healthcare-Service Needs, Psychological and Emotional Needs, and Work and Social Security Needs. However, none of the outliers were extreme, as they were all less than 3 times the interquartile range of the quartiles. Additionally, the outliers were theoretically possible and were therefore included in the analysis. The normality assumption was violated for the GAD difference, the SES difference, the SCNS difference scores in the Work and Social Security Needs and Communication and Family Needs domains, and the QoL difference scores in the Social Relationships and Environment domains. However, the paired t-test is robust to violation of this assumption as *n* > 30 (Stone, 2010).

## 3 Results

### 3.1 Sample characteristics

Participants had a variety of relationships with the cancer patients: 27.3% (*n* = 9) were partners, 21.2% (*n* = 7) were children, 21.2% (*n* = 7) were parents, and 21.2% (*n* = 7) were siblings of a cancer patient. 6.1% (*n* = 2) were in another family relationship with the cancer patient and 3% (*n* = 1) were friends of a cancer patient. More detailed information on the sample characteristics of the relatives are detailed in Table 2.

**Table 2:** Sample characteristics (*N =* 33).

Variable	*n*	%
Gender		
Men	6	18.2
Women	27	81.8
Family Status		
Single	2	6.1
Married	21	63.6
Partnership	5	15.2
Living apart	1	3.0
Divorced	3	9.1
Widowed	1	3.0
Education		
University education	11	33.3
Higher education entrance qualification	7	21.2
Secondary modern Education	9	27.3
Secondary Education	6	18.2
Cancer organism of the patient		
Intestine	3	9.1
Liver/ Gallbladder	2	6.1
Pancreas	1	3.0
Lung	7	21.2
Skin	1	3.0
Breast	4	12.1
Female Genitals	1	3.0
Male Genitals	1	3.0
Urinary organs	1	3.0
Lymphatic, haematopoietic tissue	5	15.2
Other	7	21.2
Disease stage of the patient		
Complete remission	2	6.1
Partial remission	6	18.2
Stable disease	9	27.3
Progression	9	27.3
Palliative situation	7	21.2

### 3.2 Acceptability

Initially, 35 relatives started the RELIEVE treatment. After session 1, one participant withdrew from the intervention. A second participant attended all the intervention sessions but did not fill in the surveys. This resulted in a drop-out rate of 5.71% overall and a drop-out rate of 2.86% for the intervention. The remaining 33 participants attended all stages of the procedure. At post-intervention, the CSQ had a mean sum score of 28.94 (*SD* = 2.68) which is above the cut-off score of ≥23 indicating good treatment satisfaction. More detailed information is provided in Table 3.

**Table 3:** Results of satisfaction assessment based on the CSQ.

CSQ item and answer	*n*	%
How would you rate the quality of service you received?		
Excellent (4)	11	33.3
Good (3)	20	60.6
Fair (2)	2	6.1
Poor (1)	0	0
Did you get the kind of service you wanted?		
No, definitely not (1)	0	0
No, not really (2)	0	0
Yes, generally (3)	16	48.5
Yes, definitely (4)	17	51.5
To what extent has our service met your needs?		
Almost all of my needs have been met (4)	18	54.5
Most of my needs have been met (3)	15	45.5
Only a few of my needs have been met (2)	0	0
None of my needs have been met (1)	0	0
If a friend were in need of similar help, would you recommend our service to him or her?		
No, definitely not (1)	0	0
No, I do not think so (2)	0	0
Yes, I think so (3)	7	21.2
Yes, definitely (4)	26	78.8
How satisfied are you with the amount of help you received?		
Quite dissatisfied (1)	0	0
Indifferent or mildly dissatisfied (2)	1	3.0
Mostly satisfied (3)	8	24.2
Very satisfied (4)	24	72.7
Have the services you received helped you to deal more effectively with your problems?		
Yes, they helped a great deal (4)	23	69.7
Yes, they helped somewhat (3)	10	30.3
No, they really did not help (2)	0	0
No, they seemed to make things worse (1)	0	0
In an overall, general sense, how satisfied are you with the service you received?		
Very satisfied (4)	22	66.7
Mostly satisfied (3)	11	33.3
Indifferent or mildly dissatisfied (2)	0	0
Quite dissatisfied (1)	0	0
If you were to seek help again, would you come back to our service?		
No, definitely not (1)	0	0
No, I do not think so (2)	0	0
Yes, I think so (3)	8	24.2
Yes, definitely (4)	25	75.8

CSQ-I, client, satisfaction questionnaire adapted to internet-based interventions.

### 3.3 Paired-samples *t*-tests

GAD-7 scores decreased significantly and showed a large effect size, *t*(32) = −6.65, *p* < 0.001, *d* = −1.16. PSQ Index scores decreased significantly and showed a large effect size, *t*(32) = −6.86, *p* < 0.001, *d* = −1.19. PHQ-8 scores decreased significantly and showed a large effect size, *t*(32) = −5.94, *p* < 0.001, *d* = −1.03. Scores of the QoL Physical Health domain increased significantly and showed a large effect size, *t*(32) = 4.62, *p* < 0.001, *d* = 0.81. Scores of the QoL Psychological Health domain increased significantly and showed a large effect size, *t*(32) = 4.46, *p* < 0.001, *d* = −1.19. The increase in mean scores of the QoL Social Relationships domain was not significant for the adjusted alpha level, *t*(32) = 2.01, *p* = 0.026. The increase in mean scores of the QoL Environment domain was significant and showed a moderate effect size, *t*(32) = 3.23, *p* = 0.001, *d* = 0.56. SES scores increased significantly at the original alpha level, but not significantly at the corrected alpha level, *t*(32) = 2.07, *p* = 0.024. Scores of the SCNS Healthcare Service Needs domain decreased significantly and showed a moderate effect size, *t*(32) = −3.37, *p* < 0.001, *d* = 0.59. Scores of the SCNS Psychological and Emotional Needs domain decreased significantly and showed a very large effect size, *t*(32) = −8.91, *p* < 0.001, *d* = −1.55. Scores of the SCNS Work and Social Security Needs domain decreased significantly according to the original alpha level, but were insignificant at the corrected alpha level and showed a small effect size, *t*(32) = −2.37, *p* = 0.012, *d* = −0.41. Scores of the SCNS Communication and Family Needs domain decreased significantly and showed a large effect size, *t*(32) = −6.49, *p* < 0.001, *d* = −1.13. In addition to the one-tailed *p*-values reported above, the *p*-values of the two-tailed test can be found in Table 4.

**Table 4:** Results of two-sided paired t-tests for primary outcomes.

	Paired differences								Significance
	Pre (T0)	Post (T1)	Diff.		95% CI				
	*M* (SD)	*M* (SD)	*M* (SD)	SE	Lower	Upper	*t*(32)	Cohens *d*	Two-sided *p*
GAD7	10.06 (5.18)	4.88 (3.68)	−5.18 (4.48)	0.78	−6.77	−3.59	−6.65	−1.16	<0.001
PHQ8	8.70 (5.19)	4.21 (3.94)	−4.43 (4.34)	0.76	−6.02	−2.95	−5.94	−1.03	<0.001
PSQ	0.52 (0.18)	0.35 (0.20)	−0.17 (0.14)	0.03	−0.22	−0.12	−6.86	−1.19	<0.001
QoL Phy	14.82 (2.48)	16.83 (2.43)	2.01 (2.50)	0.43	1.12	2.89	4.62	0.81	<0.001
QoL Psy	13.70 (2.43)	15.48 (3.05)	1.78 (2.29)	0.40	0.97	2.59	4.46	−1.19	<0.001
QoL Soc	15.19 (3.35)	16.00 (3.59)	0.81 (2.31)	0.40	−0.01	1.63	2.01	.	0.053
QoL Env	16.52 (1.95)	17.52 (2.16)	1.00 (1.78)	0.31	0.37	1.63	3.23	0.56	0.003
SES	27.18 (5.02)	29.09 (4.64)	1.91 (5.31)	0.92	0.03	3.79	2.06	.	0.047
SCNS Health	2.00 (0.71)	1.49 (0.64)	−0.51 (0.86)	0.15	−0.81	−0.20	−3.37	0.59	0.002
SCNS PsyEm	2.50 (0.73)	1.35 (0.48)	−1.15 (0.74)	0.13	−1.41	−0.88	−8.91	−1.55	<0.001
SCNS Work	1.59 (0.65)	1.33 (0.52)	−0.26 (0.64)	0.11	−0.49	−0.04	−2.37	−0.14	0.024
SCNS Comm	2.33 (0.92)	1.22 (0.44)	−1.10 (0.98)	0.17	−1.45	−0.76	−6.49	−1.13	<0.001

GAD-7, generalized anxiety disorder scale; PHQ-8, patient health questionnaire; PSQ-20, perceived stress questionnaire-20; SES, self-efficacy scale; SCNSP&C-G, supportive care needs survey for partners and caregivers.

## 4 Discussion

This study aimed to test the acceptability and feasibility of the RELIEVE intervention. In terms of acceptability, it was hypothesised (H1) that relatives would report high treatment satisfaction and that there would be a low drop-out rate. A total of two participants dropped out of the intervention, which can be considered to be a very low drop-out rate, especially when compared to the drop-out rates in other studies, where high rates have been reported. Treatment satisfaction was very high, which is well above the threshold for good treatment satisfaction. It should be emphasized that the participants usually chose the best two of the four response options leaving out the unsatisfactory ones (Table 2). Of course, this could also be a matter of social desirability. In terms of feasibility, it was hypothesised (H2) that the intervention would reduce anxiety, depression, stress, and need for support and increase self-efficacy and QoL when comparing pre- and post-treatment scores. The results of the t-tests showed that anxiety, depression, and stress decreased with a statistical significance with large effect sizes after completion of the intervention. The intervention did not significantly reduce work and social security needs. In terms of QoL, participants showed significantly higher QoL scores after the intervention compared to before the intervention in the domains physical health, psychological health, and environment with large to moderate effect sizes. Participants QoL did not significantly improve in the domain of social relationship. Self-efficacy did not increase significantly. Participants reported significantly lower healthcare service needs with a moderate effect size, lower psychological and emotional needs with a very large effect size, and lower communication and family needs with a large effect size.

While other group interventions did not significantly reduce anxiety and depression (Lei et al., 2023), the RELIEVE intervention was successful in reducing anxiety, depression, and stress symptoms at a statistically significant level. The findings are consistent with the literature, showing that psychoeducation is an effective tool for improving mental health and QoL in caregiving relatives (Bilgin and Özdemir, 2022; Cheng et al., 2022; Kusi et al., 2022). In addition, the treatment success of RELIEVE supports previous studies demonstrating the feasibility of combining psychoeducation with mindfulness interventions (Lei et al., 2023). Many previous interventions have targeted very small subgroups of relatives (Jones et al., 2013), which does not reflect the diverse relationships that emotionally affected relatives may have with patients. The RELIEVE study shows that interventions targeting a broader group of emotionally affected relatives are also highly effective. Offering an effective treatment to a wider target group is beneficial for widespread implementation. Furthermore, many previously studied interventions have not been tailored to the specific needs of relatives and there is a lack of standardised manuals (Kleine et al., 2019). RELIEVE provides an effective and standardised manual that was specifically designed to meet the specific needs of relatives.

Regarding the insignificant improvement in the Work and Social Security Needs domain of the SCNS, it should be noted that this domain focuses on factors that are unlikely to be influenced by the intervention, such as access to the hospital and to legal advisors or obtaining insurances for the cancer patient. The social relationships domain of the QoL questionnaire includes aspects of social support, personal relationships, and sexual activity. The non-significant findings of this domain are in contrast to some of the existing literature, which emphasises the benefits of social support in group therapy (Gray et al., 2019). On the other hand, other research has shown that group therapy did not improve social relationships outside the group therapy setting (Jones et al., 2013), which may explain this insignificant result. The marginal increase in self-efficacy is at odds with other literature that reports significant increases in self-efficacy (Cheng et al., 2022). One possible explanation for this discrepancy may be the way in which self-efficacy was measured across studies. Whilst many of the studies reviewed in the meta-analysis by Cheng et al. (2022) used self-care, cancer, or coping-specific self-efficacy questionnaires, the current study used a questionnaire measuring general self-efficacy, which may have been a too non-specific a measure. Given that 20% reported that their cancer was at a palliative stage, it may be difficult to achieve large increases in self-efficacy at a time when, despite therapeutic efforts, someone is facing an inevitable loss.

Some other limitations need to be considered. The study had a limited sample size of *N* = 33, did not include a control group, and was biased toward a more female and higher educated sample. The high percentage of women in the sample (81.8%) may limit the generalisability of the findings due to potential gender imbalance. Gender can significantly influence caregiving experiences, with research indicating that female caregivers often face higher emotional stress, physical strain, and financial challenges, potentially due to traditional caregiving roles and societal expectations. This imbalance suggests that the study’s results may not fully capture the experiences or specific needs of male caregivers. Future research should aim for a larger, more gender-balanced, and more representative sample to better understand potential sub-group differences in caregiving challenges and coping strategies. This could be achieved by implementing group interventions for relatives of cancer patients as a general offer in hospitals. The study can serve as a pilot for a comprehensive trial involving a control group. The study also did not collect data on the long-term effects of the intervention. Therefore, longitudinal studies with follow-up are needed in future research. Although the RELIEVE intervention is generally available to friends and not just family members of cancer patients, the sample in this pilot study only consisted of 3% that identified themselves as friends. Future research could explore the different needs of different subgroups of caring relatives (friends vs. family, many vs. few caring responsibilities). Additionally, differences could be explored related to the disease stage of the patients’ cancer, as the emotional impact in relatives changes throughout the disease stages and is worst during the palliative stage (Götze et al., 2016). As the burden of caregivers of patients in palliative stages is presumably worse than the burden of those caring in remission or stable stages, this could have impacted the findings and should be kept in mind when interpreting them. Relatives of patients in remission or partial remission face the challenge of uncertainty and the fear of relapse, which can lead to emotional stress. They hope for improvement but must simultaneously cope with the constant possibility of relapse. Additionally, they remain involved in supporting the patient through physical and psychological burdens. In contrast, the focus for relatives of patients in the palliative phase is on symptom relief, emotional support, and preparation for the impending loss. They must make the most of the remaining time with the patient while also dealing with grief and the inevitability of the farewell. The needs and challenges of relatives in both phases differ significantly, requiring distinct support strategies (Götze et al., 2016).

Despite these limitations, the present study fills an important research gap by investigating, for the first time, the direct effects of the RELIEVE intervention on a small, heterogeneous group of cancer patient relatives, and provides preliminary evidence of benefit. By identifying the specific needs and challenges of this group, the study provides valuable guidance for the design of future, larger and more methodologically rigorous trials. To this end, the RELIEVE programme could benefit not only caregivers but also other professionals, such as therapists, researchers, and caregiver support organizations. The programme could serve as a valuable resource for these professionals to address caregiver stress and well-being. Since the programme is already manualised in a user-friendly handbook that provides a clear structure and predefined materials like worksheets, it could also be recommended to and used by other professionals for support. It could be a practical resource for organizations aiming to promote caregiver well-being and could also serve as a foundation for further research projects.

## 5 Conclusion

The present study proposed a novel group-based intervention that was designed to meet the specific needs of cancer patient relatives. The results show that the RELIEVE intervention was well received by participants and was able to improve their levels of anxiety, depression, stress, QoL, self-efficacy, and need for support. The supportive group environment allowed participants to share their experiences and acquire useful coping and mindfulness skills. The success of this study highlights the relevance of implementing interventions that are tailored to the specific needs of cancer patient relatives. These promising results should be tested moreover in a randomized controlled setting to gain insights into the feasibility of the RELIEVE intervention.

## Data Availability

The raw data supporting the conclusions of this article will be made available by the authors, without undue reservation.
